# Influence of Raspberry and Its Dietary Fractions on the *In vitro* Activity of the Colonic Microbiota from Normal and Overweight Subjects

**DOI:** 10.1007/s11130-021-00923-6

**Published:** 2021-10-26

**Authors:** Vanesa Núñez-Gómez, Ma Jesús Periago, Inmaculada Navarro-González, Ma Piedad Campos-Cava, Nieves Baenas, Rocío González-Barrio

**Affiliations:** Grupo de Nutrición y Bromatología, Departamento de Tecnología de los Alimentos, Nutrición y Bromatología, Facultad de Veterinaria, Universidad de Murcia, CEIR Campus Mare Nostrum (CMN), Campus de Espinardo, 30100 Murcia, Spain

**Keywords:** Dietary fibre, Phenolic compounds, Urolithin, SCFAs

## Abstract

Raspberry is a source of dietary fibre and phenolic compounds, which are metabolised by the gut microbiota, resulting in the production of short chain fatty acids (SCFAs) and phenolic catabolites; but the formation of these compounds depends on the microbiota composition. The aim of this study was to investigate whether the raspberry and its fractions (phenolic extract, total and insoluble dietary fibre) affect the microbial activity depending on the body weight condition. For this, *in vitro* fermentations of raspberry fractions were carried out using faeces from normal-weight (NW) and overweight volunteers (OW) during 48 h, and phenolic catabolites and SCFAs were analysed at 0, 6, 24 and 48 h. The whole raspberry and the phenolic extract produced greater quantities of urolithins and total SCFAs when compared with fibre fractions, reaching the highest amount between 24 and 48 h. The body weight condition was an important factor, since faeces from NW led to greater production of urolithins from non-extractable phenolic compounds bound to fibre fractions, whereas in OW the urolithins production was higher from the fractions with more extractable polyphenols. In summary, the whole raspberry has been shown to have a prebiotic effect, mainly due to its phenolic compounds content rather than its fibre content.

## Introduction

Raspberry (*Rubus idaeus*) production and consumption has risen in recent years due to greater adherence to healthier diets [1], since these berries show a significant content of fibre, vitamins, minerals and phenolic compounds [2, 3]. Dietary fibre, defined as non-digestible polysaccharides, helps to prevent some diseases like colon cancer, obesity, diabetes and cardiovascular diseases, this beneficial effect being associated with its prebiotic effect [4, 5]. In addition to fibre, their phenolic compounds, mainly anthocyanins and ellagitannins, also have beneficial effects on human health (anti-cancer and neuroprotective, among others) due to their antioxidant activity [6, 7]. In most cases, phenolic compounds become attached to cell walls and act together with the dietary fibre in the large intestine, being metabolized by the gut microbiota. In this sense, when dietary fibre undergoes the colon fermentation process, SCFAs are produced, the most important being acetate, propionate and butyrate [8]. The majority of SCFAs are absorbed by colonic epithelial cells, helping to prevent colon carcinogenesis, maintaining the intestinal barrier function and promoting the development of the intestinal immune system [5, 8]. Moreover, when the raspberry-phenolic compounds, specifically ellagitannins and ellagic acid, reach the large intestine, they are metabolised by the intestinal microbiota, producing different urolithins [9, 10]. These catabolites, after their intestinal absorption, exhibit different biological activities such as antiglycative, anti-microbial, anti-cancer, anti-inflammatory, among others [7]. However, the state of the gut microbiota is very important for the performance of its essential functions in the host: fermentation of indigestible food components, removal of toxic compounds, competition with pathogens and regulation of the immune system [11, 12]. Obesity is related to dysbiosis of the microbiota, by modulation of its profile and through changes in substrate metabolism, which lead to altered catabolite production [13]. The aim of this study was to investigate the prebiotic effect of raspberry and its fractions (phenolic extract, total dietary fibre and insoluble dietary fibre), as influenced by the faecal inoculum (normal-weight and overweight subjects), in an *in vitro* fermentation model, evaluating the production of phenolic catabolites and SCFAs.

## Materials and Methods

### Samples

The whole raspberry (RAS) was obtained by a freeze-drying process, and was used to obtain its fractions, phenolic extract (PEX), total dietary fibre (TDF) and insoluble dietary fibre (IDF). In short, the fibre fractions (TDF and IDF) were extracted by enzymatic and *in vitro* digestion, followed by precipitation with or without ethanol (80%) according to the fraction, and the PEX fraction was obtained with 70%-ethanol from RAS, removing the ethanol in a rotatory evaporator [3]. The composition of the different fractions (neutral sugars, uronic acids and extractable and hydrolysable phenolic compounds) has been described by Baenas et al. [3]. In summary, all the fractions had as major compounds ellagitannins, anthocyanins and ellagic acid in their extractable fraction; besides flavonols and caffeic acid were only detected in RAS. The hydrolysable phenolic compounds were represented by ellagic acid derivatives in all the fractions, except for the PEX fraction. Indeed, the extractable phenolic compounds were more abundant in the RAS and PEX fractions, whereas the hydrolysable phenolic compounds were more abundant in the TDF and IDF fractions.

### *In vitro* Fermentation of Raspberry and Its Fractions with Human Faeces

RAS and its fractions (PEX, TDF and IDF) were fermented *in vitro* with faecal samples collected from three healthy normal-weight women (NW) and three healthy overweight women (OW), aged from 35 to 60 years old. Volunteers were non-smokers with stable food habits, had not received antibiotics for at least 3 months before the study and were free of any gastrointestinal disease. The study was approved by the Committee of Ethics of Research of the University of Murcia (Ref. No. 1434/2017) and informed written consent was obtained from each subject. Fresh faeces were collected and immediately introduced in a tube containing an AnaeroGen™ Sacket (AN35, Oxoid®, Basingstoke, Hampshire, UK) to obtain anaerobic conditions and avoid microbial modifications. The samples were processed in the first hour after deposition. The *in vitro* fermentation was performed according to the methodology described by González-Barrio et al. [14]. Samples of fresh faeces were homogenised with phosphate buffer to obtain 32% faecal slurries. Five millilitres of faecal slurry were added to 44 mL of fermentation medium at pH 7.0 and placed in a 100-mL McCartney bottle. Samples of RAS (200 mg), PEX fraction (− 2.8 mg), TDF fraction (94 mg) and IDF fraction (67 mg), previously dissolved in 1 mL of sterilised water, were added to the fermentation bottles. After these substrates had been added, the fermentation bottles were purged and then incubated for 48 h at 37 °C in a shaking bath, simulating colonic lumen conditions. Aliquots of the fermented faecal samples were collected at baseline (0 h) and after 6, 24 and 48 h and were stored at − 80 °C prior to analyses.

### Quantitative and Qualitative Analysis of Microbial Phenolic Catabolites by Liquid Chromatography

Fermented faecal samples (1 mL) were mixed with water acidified with 0.1% formic acid and passed through a C18-SPE column (Waters Corporation, Milford, Massachusetts, USA) and the compounds of interest were eluted in 1 mL of methanol [3]. The methanolic extract was analysed by HPLC–DAD (Agilent Technologies, Waldbronn, Germany), using the method described by González-Barrio et al. [15]. Urolithins and ellagic acid derivatives were identified according to their absorbance spectra, based on data previously reported [16]. Urolithins were quantified by comparison with the standard urolithin B at 305 nm and ellagic acid by comparison with the pure standard at 360 nm. The results were expressed in *µ*g mL^−1^.

### Analysis of SCFAs by Gas Chromatography

The analysis of SCFAs was performed with a gas chromatograph equipped with a flame ionization detector (GC-FID), using the protocol described by Baenas et al. [3]. Fermented faecal samples were centrifuged at room temperature for 15 min, at 16,110 *g*, and 100 *μ*L of supernatant were mixed with 650 *μ*L of 20% formic acid, methanol and 2-ethyl butyric acid as the internal standard (1/4.5/1; v/v/v). Then, the samples were vortexed for 5 min, filtered and analysed by GC-FID. Acetic acid, propionic acid and butyric acid were used as standards to identify and quantify SCFAs. The concentration of each SCFA was expressed as mmol L^−1^.

### Statistical Analysis

A two-way analysis of variance (two-way ANOVA) was performed, considering the effect of the different fractions and the body weight condition of the subjects. To determine the significance of the differences among the mean values (*p*-value < 0.05), a *post-hoc* Tukey´s test was conducted. A principal component analysis (PCA) was also performed, to correlate the metabolic activity of the microbiota with the health status of the subjects. The statistical analyses were carried out using R studio, version 3.4.3 (R Foundation for Statistical Computing, Vienna, Austria). The ellagic acid degradation and the production of urolithins and SCFAs were expressed as Δ from the baseline.

## Results and Discussion

### Microbial Phenolic Catabolites

**Fig. 1:**
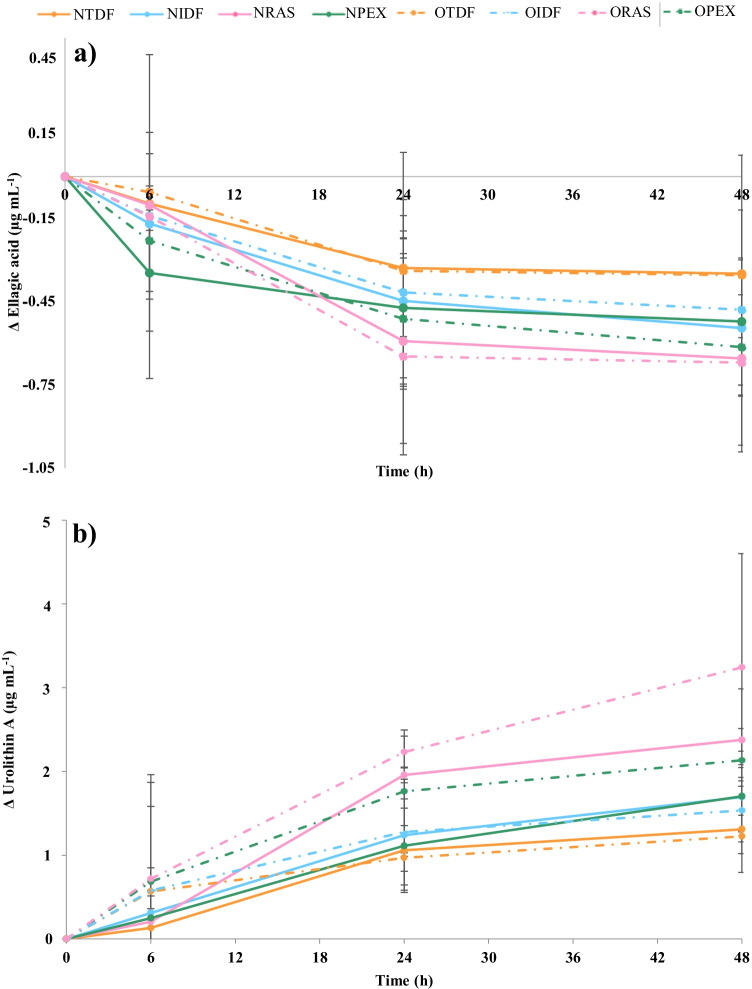
Decrease (Δ) in the content of ellagic acid (**a**) and increase (Δ) in urolithin production (µg mL^−1^) (**b**) produced by the faecal microbiota of humans (normal-weight and overweight volunteers) after 6, 24 and 48 h of *in vitro* fermentation with different substrates: NTDF (  normal-weight, total dietary fibre), NIDF (  normal-weight, insoluble dietary fibre), NRAS (  normal-weight, whole raspberry), NPEX (  normal-weight, polyphenol extract), OTDF (  overweight, total dietary fibre), OIDF (  overweight, insoluble dietary fibre), ORAS (  overweight, whole raspberry), OPEX (  overweight, polyphenol extract). Values are expressed as the means ± SD (*n* = 3)

As it has been reported previously [9, 10, 17], ellagic acid reaches the large intestine, where it can be degraded by the microbiota to urolithins. Our results show that after 24 h of *in vitro* fermentation, the bulk of the ellagic acid had been degraded by the gut microbiota to urolithin A (Fig. 1), being identified by its spectral characteristics reported by González-Barrio et al. [16]. The urolithin production rate was highest from 0 to 24 h, reaching the highest accumulation after 48 h (Fig. 1b). However, no significant differences were found in urolithin A production, neither among the different raspberry fractions, within the same group, nor between the study groups NW and OW. However, the results showed a clear tendency (Fig. 1), since the faecal incubations with RAS and PEX showed the highest ellagic acid degradation, for both groups, which resulted in higher production of urolithin A in comparison with the fermentation of dietary fibre fractions (TDF and IDF). This behaviour could be related to the differences in the content of phenolic compounds of the substrates. So, RAS had the highest amounts of ellagic acid and ellagitannins [3], which were hydrolysed to ellagic acid that was then converted into urolithins, whereas TDF fraction showed the lowest ellagic acid degradation rate, and consequently the lowest urolithin production due to the lowest content of extractable ellagitannins and ellagic acid [3]. Moreover, this fraction had the highest amount of hydrolysable phenolic compounds, and as non-extractable phenolic compounds, their metabolisation by the microbiota may have been hampered since they bind to cell walls. So, the higher production of urolithins from RAS and PEX fractions appeared to be associated to the bioaccessibility of their precursors, because the extractable phenolic compounds can be rapidly metabolised by the microbiota. It is also notable that the urolithin production (Fig. 1b) was similar for RAS and PEX during the first 6 h, but after 24 h differences were apparent, might be due to the distinct compositions of the two fractions. PEX fraction only contained extractable ellagic acid, while ellagic acid in RAS was also present attached to the matrix and, therefore, it might be released from the cell walls and promote urolithin production over a longer period.

As far as we know, only Inada et al. [18] analysed *in vivo* the production of urolithins after ingestion of a berry powder in NW and OW subjects, reporting similar results that are in agreement with our findings, without significant differences between the weight condition. However, a tendency was also observed related to the body weight condition. It is important to highlight that the fecal inoculum of NW produced more urolithins from the fibre fractions (TDF and IDF) than those of OW. By contrast, the fermentation with the fecal inoculum of OW produced more urolithins from the RAS and PEX fractions, probably due to their amount of extractable phenolic compounds (Fig. 1b). Hence, our results show that when the bulk of the phenolic compounds were extractable, the microbiota of OW was able to produce a greater quantity of urolithins than the microbiota from NW. However, for the fractions with more hydrolysable phenolic compounds (TDF and IDF), the microbiota from OW was less able to bio-transform them into urolithins.

### SCFAs Production

The potential prebiotic effect of the different substrates assayed was measured by evaluation of the SCFAs produced by the gut microbiota. As we can see in Table 1, acetate was the major SCFA produced, for both NW and OW groups, since acetate is recognized as the main SCFA produced by the intestinal bacteria [19, 20]. RAS produced the highest amounts of acetate, propionate and total SCFAs, followed by the PEX fraction. These results reveal that not only dietary fibre from raspberry exhibits a prebiotic effect [5] but also its phenolic compounds (Table 1) may be considered as potential prebiotics, showing a synergistic effect [21]. On the contrary, the fibre fractions (TDF and IDF) produced the lowest amount of total SCFAs, which indicates a lower prebiotic effect compared with RAS and PEX fraction. The prebiotic effect of fibre depends on its solubility, and TDF fraction has a low content of soluble dietary fibre (only 28%, mainly pectins and soluble hemicellulose), whereas IDF fraction contains the non-fermentable carbohydrates (mostly cellulose) [3]. For this reason, the fermentation of TDF fraction led to a higher formation of SCFAs than IDF fraction. The butyrate content showed the smallest differences when comparing TDF and IDF with RAS and PEX, since butyrate is mainly produced from non-digestible polysaccharides, and therefore, in the fibre fractions the production was equalised to the other two fractions [22].

**Table 1:** Increase (Δ) in short-chain fatty acids (SCFAs) (mmol L^−1^) (acetate, propionate, butyrate and total) produced by human faecal microbiota after 6, 24 and 48 h of *in vitro* fermentation with different substrates: RAS (whole raspberry), PEX (polyphenol extract), TDF (total dietary fibre) and IDF (insoluble dietary fibre). The faecal samples were obtained from NW (normal-weight) and OW (overweight) volunteers

	RAS			PEX			TDF			IDF		
	6 h	24 h	48 h	6 h	24 h	48 h	6 h	24 h	48 h	6 h	24 h	48 h
NW												
Acetate	12.5 ± 1.6^a^	15.9 ± 5.3^a^	14.5 ± 5.5 ^a^	6.4 ± 1.3*^b^	9.0 ± 6.3 ^ab^	13.3 ± 2.4^ab^	4.6 ± 0.9^b^	9.4 ± 2.6 ^ab^	9.3 ± 4.4^ab^	4.1 ± 1.1^b^	6.4 ± 0.6 ^b^	6.8 ± 0.9*^b^
Propionate	3.1 ± 0.7*^a^	3.7 ± 0.7 ^a^	3.6 ± 0.5 ^a^	1.3 ± 0.1*^b^	1.9 ± 1.0 ^b^	2.7 ± 0.6 ^ab^	1.0 ± 0.4^b^	1.8 ± 1.2 ^b^	1.9 ± 1.1 ^b^	1.0 ± 0.4^b^	1.4 ± 0.5 ^b^	1.6 ± 0.7^b^
Butyrate	1.7 ± 0.2	2.5 ± 0.4 ^a^	2.6 ± 1.0	1.4 ± 0.5	2.1 ± 1.1 ^ab^	2.8 ± 0.2	1.5 ± 0.6	1.9 ± 0.5 ^ab^	2.1 ± 0.6	1.4 ± 0.1	1.7 ± 0.5 ^b^	1.9 ± 0.6
Total	17.3 ± 1.3^a^	22.0 ± 5.8	20.6 ± 6.7 ^a^	9.1 ± 1.9*^b^	12.9 ± 8.4	18.8 ± 1.9^ab^	7.1 ± 1.2^b^	13.1 ± 4.0	13.2 ± 5.6^ab^	6.6 ± 1.4^b^	9.5 ± 0.4	10.4 ± 1.8^b^
OW												
Acetate	9.9 ± 5.5 ^ab^	22.2 ± 5.9^a^	22.1 ± 9.1	15.0 ± 2.6*^a^	21.1 ± 7.6^a^	20.4 ± 10.6	5.0 ± 0.4^b^	9.1 ± 2.1 ^b^	9.3 ± 1.4	4.1 ± 2.0^b^	8.3 ± 2.7 ^b^	9.0 ± 0.1*
Propionate	0.9 ± 0.9*^ab^	3.6 ± 1.2 ^a^	3.9 ± 2.2	1.7 ± 0.3*^a^	3.5 ± 1.5 ^a^	3.6 ± 2.4	0.7 ± 0.2 ^b^	1.5 ± 0.5 ^b^	1.6 ± 0.4	0.7 ± 0.4 ^b^	1.2 ± 0.5 ^b^	1.6 ± 0.6
Butyrate	0.9 ± 1.0 ^b^	2.4 ± 0.8 ^ab^	2.5 ± 0.8	2.3 ± 0.2^a^	3.0 ± 0.7 ^a^	2.9 ± 0.6	1.5 ± 0.2^ab^	1.8 ± 0.3 ^b^	1.9 ± 0.4	1.0 ± 0.4^ab^	1.9 ± 0.3 ^b^	1.9 ± 0.5
Total	11.7 ± 7.2^ab^	28.2 ± 7.8^a^	28.5 ± 12.0	19.0 ± 3.1*^a^	27.6 ± 9.7^a^	26.9 ± 13.5	7.2 ± 0.5^b^	12.4 ± 2.6^ab^	12.8 ± 1.6	5.8 ± 2.5^b^	11.3 ± 2.8^b^	12.4 ± 0.3

Values are expressed as the means ± SD (*n* = 3). Different letters (a–c) indicate significant differences (*p* < 0.05) among the fractions at the same time. *Indicates significant differences (*p* < 0.05) between normal-weight and overweight volunteers

It is known that overweightness leads to a higher amount of SCFAs in faeces because the intestinal microbiota from OW people showed a better ability to produce SCFAs [20]. However, we did not observe a clear effect, and the amounts of SCFAs were mainly related to the fermentable substrate or sample. The propionate production from RAS was higher in the NW at 6 h. On the other hand, the production of acetate, propionate and total SCFAs from the PEX fraction was higher in the OW at 6 h, and acetate production from the IDF fraction was higher in OW at 48 h. This could be due to the high production of urolithins in the OW, since urolithins have antimicrobial activity [23] and can cause a decrease in the gut microbial activity leading to a reduction in SCFAs production.

**Fig. 2:**
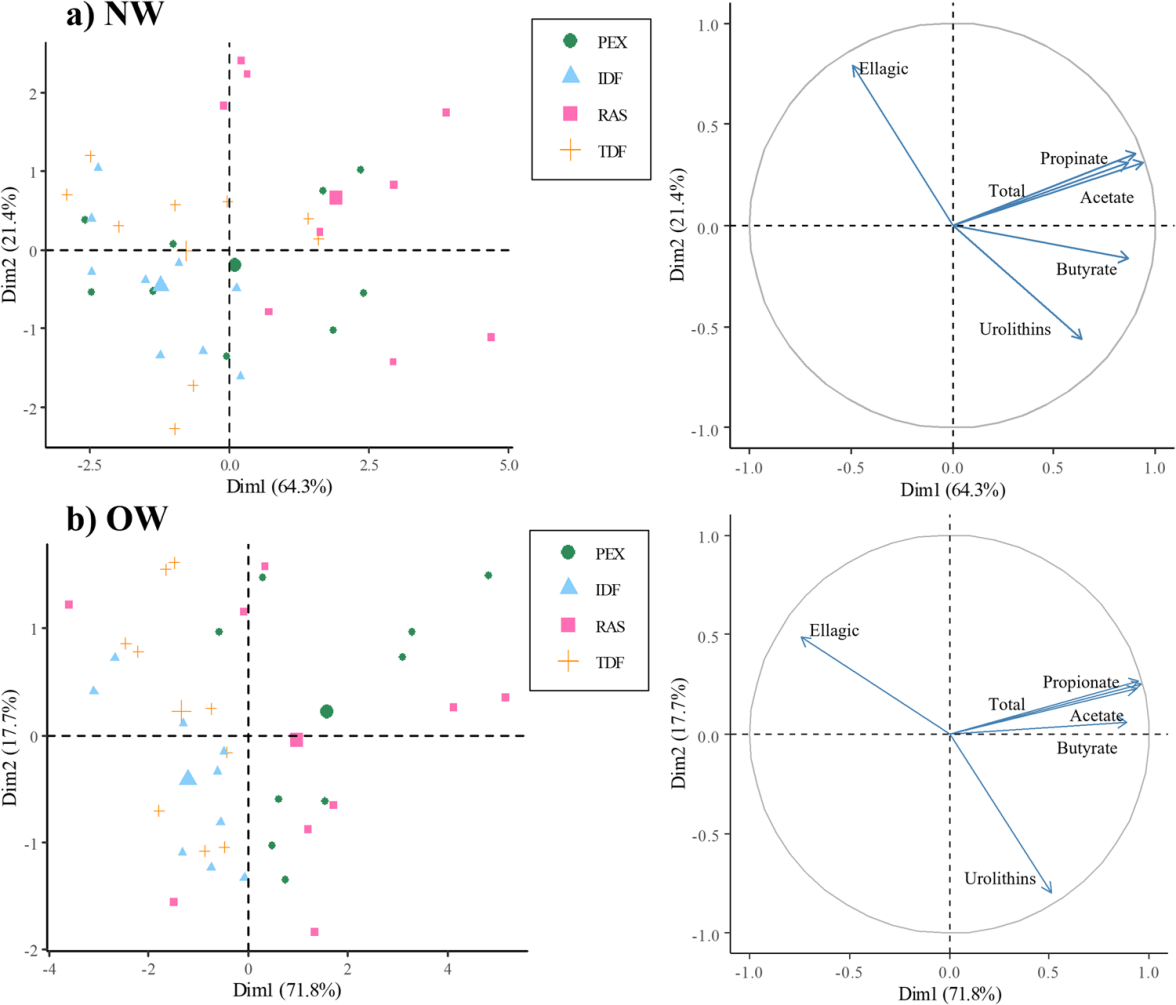
Principal component analysis (PCA) showing individual distribution (on the left) and variables distribution (on the right) for selected variables (Δ ellagic acid, urolithin A, acetate, propionate, butyrate and total SCFAs) in normal-weight volunteers (**a** NW) and overweight volunteers (**b** OW). Different colours show different fractions: PEX (  polyphenol extract), IDF (  insoluble dietary fibre), RAS (  whole raspberry), TDF (  total dietary fibre)

Multivariate statistical analysis was used to determine the relationship between the substrates and the metabolites produced during fermentation. In this sense, a PCA was performed separately for NW and OW (Fig. 2). The representation of the subjects is shown on the left side of the figure, and that of the variables on the right. The analyses identified six dimensions (Dim), the first two explaining 85.7% of the total variance for NW and 89.5% for OW. The contents of individual (acetate, propionate, butyrate) acids, total SCFAs and urolithins were positively correlated with Dim 1, in both groups, whereas ellagic acid was negatively correlated with this dimension. Dim 2 represented the formation of urolithins, being negatively correlated with butyrate and urolithins for NW but only with urolithins for OW. It is noteworthy that the samples of the different fractions of raspberry were clearly separated, but in a different way depending on the body weight condition. Figure 2a shows that NW RAS was the substrate that gave rise to the greatest formation of beneficial metabolites and catabolites from the activity of the microbiota. Contrastingly, in OW (Fig. 2b) PEX fraction led to the greatest formation of these compounds, followed by RAS. In addition, these results highlight that there was a relationship between urolithins and butyrate production in the NW but not in the OW. This relationship could be explained by the greater ability of the NW to metabolise phenolic compounds from the non-digestible fractions (TDF and IDF), leading to higher production of urolithins and butyrate at the same time. These results indicate that body weight and the related condition of the microbiota could determine the beneficial effects of prebiotic functional food and functional ingredients.

## Conclusions

The whole raspberry exhibits a prebiotic effect which is mainly due to its phenolic compounds content and not so much to its fibre content. However, the phenolic compounds associated with dietary fibre fractions also contribute to the activity of the gut microbiota. These results are interesting for the development of prebiotic functional ingredients, taking into consideration that their beneficial effects depend mainly on the ingredient composition but may be also influenced by the body weight condition, since the microbiota determine the metabolites and catabolites formed in the colon. Further studies of the changes in the individual groups comprising the microbiota are required.

## Data Availability

The datasets generated during the current study are available from the corresponding author on reasonable request.

## Abbreviations

RAS: Raspberry
PEX: Polyphenol extract
TDF: Total dietary fibre
IDF: Insoluble dietary fibre
NW: Normal-weight
OW: Overweight
SCFAs: Short chain fatty acids
GC-FID: Gas chromatography coupled to a flame ionization detector
HPLC–DAD: High performance liquid chromatography with a diode array detector
PCA: Principal components analysis

## References

[1] Vázquez-González M, Fernández-Prior Á, Bermúdez Oria A (2020). Utilization of strawberry and raspberry waste for the extraction of bioactive compounds by deep eutectic solvents. LWT.

[2] Paredes-López O, Cervantes-Ceja ML, Vigna-Pérez M, Hernández-Pérez T (2010). Berries: improving human health and healthy aging, and promoting quality life-a review. Plant Foods Hum Nutr.

[3] Baenas N, Nuñez-Gómez V, Navarro-González I, Sánchez-Martínez L, García-Alonso J, Periago MJ, Gozález-Barrio R (2020). Raspberry dietary fibre: chemical properties, functional evaluation and prebiotic *in vitro* effect. LWT.

[4] Fuller S, Beck E, Salman H, Tapsell L (2016). New horizons for the study of dietary fiber and health: a review. Plant Foods Hum Nutr.

[5] Hijová E, Bertková I, Štofilová J (2019). Dietary fibre as prebiotics in nutrition. Cent Eur J Public Heal.

[6] Szajdek A, Borowska EJ (2008). Bioactive compounds and health-promoting properties of berry fruits: a review. Plant Foods Hum Nutr.

[7] Jiang Y, Fang Z, Leonard W, Zhang P (2021). Phenolic compounds in Lycium berry: composition, health benefits and industrial applications. J Funct Foods.

[8] Wang M, Wichienchot S, He X, Fu X, Huang Q (2019). *In vitro* colonic fermentation of dietary fibers: fermentation rate, short-chain fatty acid production and changes in microbiota. Trends Food Sci Technol.

[9] Kujawska M, Jodynis-Liebert J (2020). Potential of the ellagic acid-derived gut microbiota metabolite—Urolithin A in gastrointestinal protection. World J Gastroenterol.

[10] González-Barrio R, Borges G, Mullen W, Crozier A (2010). Bioavailability of anthocyanins and ellagitannins following consumption of raspberries by healthy humans and subjects with an ileostomy. J Agric Food Chem.

[11] Cunningham AL, Stephens JW, Harris DA (2021). A review on gut microbiota: a central factor in the pathophysiology of obesity. Lipids Heal Dis.

[12] Heintz-Buschart A, Wilmes P (2018). Human gut microbiome: function matters. Trends Microbiol.

[13] Liu R, Hong J, Xu X (2017). Gut microbiome and serum metabolome alterations in obesity and after weight-loss intervention. Nat Med.

[14] González-Barrio R, Edwards CA, Crozier A (2011). Colonic catabolism of ellagitannins, ellagic acid, and raspberry anthocyanins: *In vivo* and *in vitro* studies. Drug Metab Dispos.

[15] González-Barrio R, Periago MJ, Luna-Recio C, García-Alonso FJ, Navarro-González I (2018). Chemical composition of the edible flowers, pansy (*Viola**wittrockiana*) and snapdragon (*Antirrhinum**majus*) as new sources of bioactive compounds. Food Chem.

[16] González-Barrio R, Truchado P, Ito H, Espín JC, Tomás-Barberán F (2011). UV and MS identification of urolithins and nasutins, the bioavailable metabolites of ellagitannins and ellagic acid in different mammals. J Agric Food Chem.

[17] Hao Y, Yang J, Cui J, Fan Y, Li N, Wang C, Liu Y, Dong Y (2021). Stability and mechanism of phenolic compounds from raspberry extract under *in vitro* gastrointestinal digestion. LWT.

[18] Inada KOP, Tomás-Barberán FA, Perrone D, Monteiro M (2019). Metabolism of ellagitannins from jabuticaba (*Myrciaria jaboticaba*) in normoweight, overweight and obese Brazilians: unexpected laxative effects influence urolithins urinary excretion and metabotype distribution. J Funct Foods.

[19] Karimi R, Azizi MH, Sahari MA, Kazem AE (2020). *In vitro* fermentation profile of soluble dietary fibers obtained by different enzymatic extractions from barley bran. Bioact Carbohydr Diet Fibre.

[20] Nogacka AM, Salazar N, Arboleya S (2020). *In vitro* evaluation of different prebiotics on the modulation of gut microbiota composition and function in morbid obese and normal-weight subjects. Int J Mol Sci.

[21] Alves-Santos AM, Sugizaki CSA, Lima GC, Naves MMV (2020). Prebiotic effect of dietary polyphenols: a systematic review. J Funct Foods.

[22] Bas-Bellver C, Andrés C, Seguí L (2020). Valorization of persimmon and blueberry byproducts to obtain functional powders. *In vitro* digestion and fermentation by gut microbiota. J Agric Food Chem.

[23] Singh R, Chandrashekharappa S, Vemula PK, Haribabu B, Rao-Jala V (2020). Microbial metabolite urolithin b inhibits recombinant human monoamine oxidase a enzyme. Metabolites.

